# A Spontaneous, Recurrent Mutation in Divalent Metal Transporter-1 Exposes a Calcium Entry Pathway

**DOI:** 10.1371/journal.pbio.0020050

**Published:** 2004-03-16

**Authors:** Haoxing Xu, Jie Jin, Louis J DeFelice, Nancy C Andrews, David E Clapham

**Affiliations:** **1**Howard Hughes Medical Institute, Children's HospitalHarvard Medical School, Boston, MassachusettsUnited States of America; **2**Department of Pharmacology, Vanderbilt University Medical CenterNashville, TennesseeUnited States of America

## Abstract

Divalent metal transporter-1 (DMT1/DCT1/Nramp2) is the major Fe^2+^ transporter mediating cellular iron uptake in mammals. Phenotypic analyses of animals with spontaneous mutations in *DMT1* indicate that it functions at two distinct sites, transporting dietary iron across the apical membrane of intestinal absorptive cells, and transporting endosomal iron released from transferrin into the cytoplasm of erythroid precursors. DMT1 also acts as a proton-dependent transporter for other heavy metal ions including Mn^2+^, Co^2+^, and Cu^2^, but not for Mg^2+^ or Ca^2+^. A unique mutation in *DMT1,* G185R, has occurred spontaneously on two occasions in microcytic *(mk)* mice and once in Belgrade *(b)* rats. This mutation severely impairs the iron transport capability of DMT1, leading to systemic iron deficiency and anemia. The repeated occurrence of the G185R mutation cannot readily be explained by hypermutability of the gene. Here we show that G185R mutant DMT1 exhibits a new, constitutive Ca^2+^ permeability, suggesting a gain of function that contributes to remutation and the *mk* and *b* phenotypes.

## Abstract 2

David Clapham and colleagues offer new evidence that blurs the line between ion transporters and channels

## Introduction

Spontaneous mutations in mice and rats have provided important information about mammalian iron homeostasis (reviewed in Andrews 2000). Interestingly, three independent, autosomal recessive mutants have been shown to have the same amino acid substitution in a key iron transport molecule. Two strains of mutant microcytic *(mk)* mice (MK/ReJ-*mk*, SEC/1ReJ-*mk*) and Belgrade (*b*) rats have severe iron deficiency attributable to a G185R mutation in divalent metal transporter-1 (DMT1) (Fleming et al. 1997; Andrews 2000). Based on the phenotypes of these animals and the properties of DMT1 detailed below, we and others concluded that DMT1 is essential for intestinal absorption of Fe^2+^ and for unloading of transferrin-derived iron from transferrin cycle endosomes (Fleming et al. 1997, 1998; Gunshin et al. 1997; Picard et al. 2000). It is intriguing that no other *DMT1* mutations have been described in mammals, and no features of the DNA sequence suggest that the G185 codon would be hypermutable in two species. We speculated that a novel characteristic of the G185R DMT1 protein might account for this remarkable pattern of remutation.

**Figure 1:**
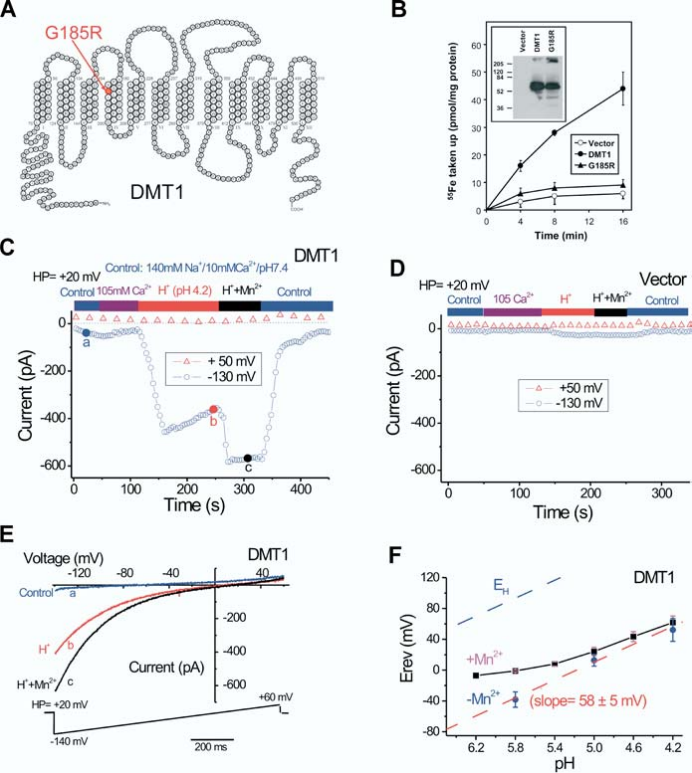
Wild-Type DMT1-Expressing Cells Exhibit a Proton Current and a Proton-Dependent Mn^2+^-Induced Current (A) The G185R mutation is in the fourth of 12 putative TM domains in both mouse (shown) and rat DMT1 proteins. (B) ^55^Fe^2+^ uptake was greatly reduced for G185R in comparison to wild-type DMT1, although the protein expression levels were comparable (inset). (C–E) Representative currents induced by protons (pH 4.2) and Mn^2+^ (100 μM) at +50 mV (open triangles; some of the datapoints have been removed for clarity) and −130 mV (open circles) in a wild-type DMT1-transfected CHO-K1 cell. Whole-cell currents were elicited by repeated voltage ramps (−140 to +60 mV, 1,000 ms), shown in (E), with a 4 s interval between ramps. Holding potential (HP) was +20 mV. Neither control solution (10mM Ca^2+^/140 mM Na^+^/[pH7.4]) nor isotonic Ca^2+^ (105 mM) solution induced significant current. Representative I-V relations are shown in (E). Current responses from a vector (pTracer)-transfected cell are shown in (D). (F) pH-dependence of the E_rev_ of the wild-type DMT1 current in the presence or absence of 300 μM [Mn^2+^]_o_. In the absence of Mn^2+^, the pH dependence of the E_rev_ can be fitted by a line with a slope 58 mV/pH unit. In the presence of 300 μM Mn^2+^, the relationship was nonlinear, especially at higher pH. E_H_, H^+^ equilibrium potential. Note that the currents were not leak-subtracted.

Trace metal ions including Fe^2+^, Mn^2+^, Cu^2+^, Zn^2+^, and Co^2+^ are required cofactors for many essential cellular enzymes. They cannot cross the plasma membrane through simple diffusion, and active uptake requires specific transporters. DMT1 is the only molecule known to mediate cellular iron uptake in higher eukaryotes. It is structurally unrelated to known Zn^2+^ and Cu^2+^ transporters, but DMT1 can transport those and other divalent metal ions (Gunshin et al. 1997), and it appears to be the major mammalian Mn^2+^ transporter (Chua and Morgan 1997). DMT1 is predicted to have 12 transmembrane (TM) segments (Figure 1A). It is expressed on the apical brush border of the proximal duodenum (Canonne-Hergaux et al. 1999) and in transferrin cycle endosomes (Su et al. 1998; Gruenheid et al. 1999). It appears to function by coupling a metal entry pathway to a downhill proton gradient, taking advantage of the acidic pH in both of those sites. An earlier study proposed a 1:1 stoichiometry of metal ion and proton cotransport (Gunshin et al. 1997).

Ca^2+^ is not a measurable substrate for wild-type DMT1 (Gunshin et al. 1997; Tandy et al. 2000), even though it is at least 1,000 times more abundant in plasma than trace metals. Surprisingly, we found that the G185R mutation (Figure 1A) dramatically increases the Ca^2+^-permeability of DMT1, functionally converting DMT1 into a Ca^2+^ channel. In light of the important and ubiquitous role of Ca^2+^ in cell signaling (Berridge et al. 2003), this gain of function offers a likely explanation for the remutation.

Interpretations of recent structural data have already suggested that permeation pathways exist within some transporters (Hirai et al. 2002), blurring the distinction between transporters and ion channels (DeFelice and Blakely 1996). Our finding, that a single amino acid substitution in a presumed transporter can expose a channel pathway, strongly supports this notion and provides new insight into what must be viewed as a continuum between transporter and channel activities.

## Results

We studied wild-type DMT1 and the G185R mutant proteins by whole-cell patch–clamp in transiently expressing CHO-K1 and HEK-293T cells and in doxycycline-inducible DMT1-HEK-On and G185R-HEK-On cells. Consistent with previous studies, DMT1 expression significantly increased cellular ^55^Fe^2+^ uptake at low pH (Figure 1B). As reported in *Xenopu*s oocytes (Gunshin et al. 1997), reduction of extra-cellular pH in the absence of metal (nominal free [Fe^2+^]_o_ of approximately 0.05 μM) induced large inward currents in DMT1-expressing cells (Figure 1C and 1D). This current is referred to as a substrate-free “leak” pathway and is representative of “drive-slip” phenomena seen in DMT1 and a related yeast metal transporter, SMF1p (Sacher et al. 2001), as well as many other transporters (Nelson et al. 2002). Because we found that protons also activated an endogenous diisothiocyanostilbene 2,2-disolphonic acid (DIDS)-sensitive anion conductance (unpublished data) that was strongly outwardly rectifying (Figure S1), we used SO_4_^2–^ to replace most of the Cl^–^ ([Cl^–^]_o _= 5 mM) in low-pH bath solutions. With elimination of the background Cl^–^ current, the proton-evoked current was inwardly rectifying (hyperbolic) (Figure 1E).

The large proton-induced current caused significant DMT1-specific intracellular acidification (Gunshin et al. 1997). In whole-cell recordings of DMT1 currents, we routinely observed slow inactivation (or decay) after a proton-induced current reached its peak (see Figure 1C). While the extent of the slow inactivation varied from cell to cell, it usually reached a relative steady state within 100 s. Addition of 100 μM Fe^2+^ (data not shown) or Mn^2+^ induced an additional current with less pronounced slow inactivation (Figure 1C). Because Fe^2+^ is readily oxidized to Fe^3+^ in the absence of substantial concentrations of reducing agents (e.g., ascorbate), and Fe^3+^ is not transported by DMT1 (Gunshin et al. 1997; Picard et al. 2000), we have used Mn^2+^ as an Fe^2+^ surrogate since both metals induced similar currents (Gunshin et al. 1997; unpublished data). The observed Mn^2+^ deficiency of *b* rats in vivo (Chua and Morgan 1997) also supports its use in this role.

H^+^ alone or H^+^/Mn^2+^ induce distinct currents in DMT1. No significant voltage- or time-dependent fast inactivation was seen when the DMT1-mediated H^+^/Mn^2+^ current (I_DMT1_) was recorded (Figure S2). The amplitude of additional Mn^2+^-induced current was dependent on [Mn^2+^]_o_, with a measurable response at [Mn^2+^]_o_ < 1 μM (pH 4.2). In the presence of 100 μM Mn^2+^ (pH 4.2), the additional Mn^2+^-induced current was typically half the amplitude of the proton-induced current. Addition of Mn^2+^ alone (100 μM) at pH 7.4 did not induce any additional current. Since H^+^ or H^+^/Mn^2+^ induced two currents with distinct kinetics in DMT1-expressing cells, the underlying charge-carrying ion species and their relative contributions to the macroscopic currents were investigated. We monitored the reversal potential (E_rev_) and the current amplitude in ion-substitution experiments. Replacement of Na^+^ with *N*-methyl-D-glucamine (NMDG^+^) did not significantly change the E_rev_ of H^+^ or H^+^/Mn^2+^-induced currents, although the net current amplitude was slightly increased (Figure S3). On the other hand, the current amplitude (data not shown) and E_rev_ of the proton current were strongly affected by [H^+^]_o_ (see Figure 1F). The slope of E_rev_ versus pH was 58 mV/decade, is consistent with an H^+^-permeable pore. The large positive displacement in E_rev_ from E_H_ (see Figure 1F) may result in part from leak and capacitance-charging, but the carrier mechanism is not well understood.

In contrast, when Mn^2+^ was introduced, the slope of the curve fitted to E_rev_ versus pH deviated considerably from the theoretical slope for a H^+^-permeable electrode (see Figure 1F). Replacement of Na^+^ by NMDG^+^ did not significantly affect the Mn^2+^-induced response (see Figure S3). Our interpretation of this deviation is that DMT1 transport stoichiometry is variable (Chen et al. 1999; Sacher et al. 2001; Adams and DeFelice 2002) or has a fixed but very low permeation ratio (P_Mn_/P_H_) (Hodgkin and Horowicz 1959). P_Mn_/P_H_ can be estimated from the slope of E_rev_ versus pH based on an extended Goldman–Hodgkin–Katz equation (Lewis 1979) with two permeable ions (H^+^ and Mn^2+^). At pH 4.2, the slope of E_rev_ versus pH did not differ significantly with or without Mn^2+^(see Figure 1F). Therefore, we estimate that at pH 4.2 the contribution of H^+^ to I_DMT1_ is much larger than that of Fe^2+^/Mn^2+^ (P_Mn_/P_H _< 0.01), in contrast to the 1:1 stoichiometry proposed previously (Gunshin et al. 1997). Importantly, no Ca^2+^ permeability was observed, even in isotonic (105 mM) Ca^2+^ solution (see Figure 1C).

**Figure 2:**
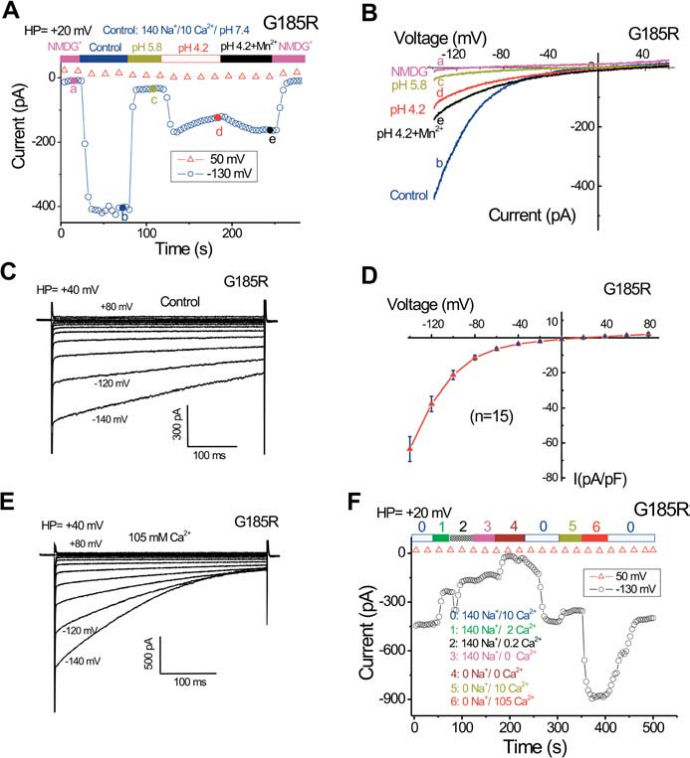
G185R-Expressing Cells Display a Constitutive [Ca^2+^]_o_-Dependent Cationic Current (A–B) Large inward currents were evoked by control solution (10mM Ca^2+^/140 mM Na^+^ [pH 7.4]) in G185R-transfected cells. The current was inhibited by lowering the solution pH to 5.8 without altering other ions. Further reducing the pH to 4.2 induced I_DMT1_-like current (enhanced by adding 100 μM Mn^2+^). No significant inward current was seen in NMDG^+^ (Na^+^-free, Ca^2+^-free) solution. (C) Time- and voltage-dependent kinetics of I_G185R_ recorded in control solution in response to voltage steps. (D) Current densities (mean ± SEM, *n* = 15) of I_G185R_ in control solution mea-sured at various voltages and normalized by cell capacitance. (E) Time- and voltage-dependent kinetics of I_G185R_ in the presence of 105 mM Ca^2+^. (F) Ca^2+^ is more permeant than Na^+^ in G185R-expressing cells.

In G185R-expressing cells, we observed a large inward current in control bath solution (10 mM Ca^2+^ and 140 mM Na^+^) at pH 7.4 (Figure 2A), though no significant current was detected with wild-type DMT1 under similar conditions (see Figure 1E). This inward current mediated by G185R mutant DMT1 (I_G185R_) was stable over minutes with no slow inactivation (see Figure 2A), in contrast to the DMT1-mediated proton current (see Figure 1C). We observed I_G185R_ in more than 85% of enhanced green fluorescent protein (EGFP)-positive cells transfected with the pTracer-G185R construct and in stable, doxycycline-induced G185R-HEK-On cells, but never in cells transfected with wild-type *DMT1* (Figure 1C) or with 30 *DMT1* mutations at other positions (*n* > 300 cells; unpublished data). The inwardly rectifying current was cationic, since Ca^2+^ and Na^+^ substitution by NMDG^+^ completely abrogated the current (see Figure 2A and 2B). The current and rectification profiles were not significantly changed when ATP and Mg^2+^ were omitted from the intracellular solution, or when Na^+^ or K^+^ replaced Cs^+^ as the primary intracellular cation.

We found that low pH strongly inhibited I_G185R_ (by approximately 90% at pH 5.8; Figure 2A), in contrast to both wild-type DMT1 currents, which were activated at low pH. However, further reduction to pH 4.2 revealed a current (Figure 2A and 2B) that was similar to the proton current of wild-type DMT1. Addition of Mn^2+^ at pH 4.2 enhanced the inward current, as with wild-type DMT1 (Figure 2A and 2B). The proton current and Mn^2+^-induced response displayed similar patterns of inactivation and further activation as in wild-type DMT1-transfected cells, but both currents were much smaller than their wild-type counterparts. Consistent with this result and our previous uptake studies (Su et al. 1998), we found that G185R cells had much lower Fe^2+^ uptake (approximately 10% measured at 16 min) compared to wild-type DMT1 at similar protein expression levels (see Figure 1B).

I_G185R_ rectified more steeply with voltage than I_DMT1_, probably due to pronounced time- and voltage-dependent fast inactivation (Figure 2C; see Figure S2 for comparison). Fast inactivation was enhanced when [Ca^2+^]_o _was increased to 105 mM (Figure 2E), strengthening the notion that I_G185R_ was fundamentally distinct from the currents mediated by wild-type DMT1. In control bath solution (10 mM Ca^2+^, 140 mM Na^+^ [pH 7.4]), I_G185R _was 64 ± 7 pA/pF at −140 mV (mean ± SEM, *n* = 15; Figure 2D) compared to less than 2 pA/pF in mock and DMT1-transfected cells. I_G185R_ reversed at approximately +20 mV with very little current above 0 mV (Figure 2D), whereas the E_rev_ of I_DMT1_ was approximately +50 mV at pH 4.2. The dependence of I_G185R_ on holding potential was also distinct from I_DMT1_ (see below).

**Figure 3:**
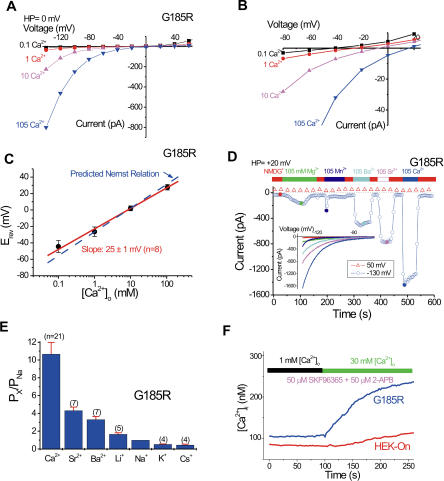
Ca^2+^ Permeability of I_G185R_ (A) Whole-cell I-V relations in the presence of [Ca^2+^]_o_ are indicated. (B) Enlarged view of (A) to show the E_rev_ measurement. (C) [Ca^2+^]_o_ dependence of E_rev_. The slope was fit by linear regression to 25 mV per decade, close to the 29 mV per decade predicted for a Ca^2+^-selective electrode (dotted line). (D) Currents through G185R in various isotonic divalent solutions. I-Vs are shown in the inset. Note that currents induced by isotonic Mg^2+^ and Mn^2+^ were transient. (E) Relative permeability of various divalent and monovalent cations**.** The reversal potentials of I_G185R_ in 10 mM test divalent cations were measured under bi-ionic conditions as described in Materials and Methods. The permeability was calculated using Equations 1 and 2. (F) [Ca^2+^]_i_ changes estimated by Fura-2 fluorescence in response to an elevation of [Ca^2+^]_o_ from 1 to 30 mM. The results were averaged from five (HEK-On) and seven (G185R) independent experiments (*n* = 3–13 cells each). To minimize potential endogenous depletion-activated and/or TRP-mediated Ca^2+^ influx, cells were bathed in the presence of 50 μM SKF96365 and 50 μM 2-APB. The F340/F380 ratio was recorded and converted into estimated [Ca^2+^]_i_ based on an ionomycin-induced Ca^2+^ calibration.

**Figure 4:**
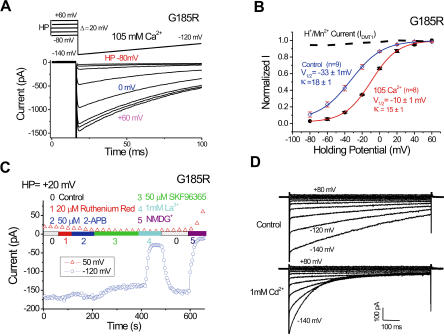
Voltage Dependence and Pharmacological Properties of I_G185R_ (A) Whole-cell currents recorded in 105 mM [Ca^2+^]_o_ were dependent on holding potential before the voltage ramps (−140 to −120 mV shown). For clarity, only the first 20 ms of the 4 s-long holding potential is shown. (B) Voltage dependence of I_G185R _in control solution and 105 mM [Ca^2+^]_o_. I_DMT1_ (dotted line) exhibited no depen-dence on the holding potential. Abbreviations: V_1/2_ , half activation voltage. κ, slope factor. (C and D) Sensitivity of I_G185R _to various pharmacological agents and cation channel blockers. I_G185R_ was relatively insensitive to RR, 2-APB, or SKF96365, but was blocked by 1mM La^3+^ or Cd^2+^ (D).

We next investigated the cation selectivity of I_G185R_. The amplitude of I_G185R_ was strongly dependent on [Ca^2+^]_o_ (Figure 2F). With 10 mM Ca^2+^ in the bath, replacement of 140 mM NMDG^+^ by 140 mM Na^+^ only slightly (by approximately 15%) increased the current, indicating that Ca^2+^ permeated the plasma membrane of G185R-transfected cells much more readily than Na^+^. As shown in Figure 3A and 3B, increasing [Ca^2+^]_o_ not only augmented the current amplitude but also shifted E_rev_ toward depolarized potentials. The slope of this shift (25 mV per decade) was close to the slope of 29 mV per decade predicted by the Nernst equation for a Ca^2+^-selective electrode (Figure 3C). The relative permeability of various divalent cations was studied under bi-ionic conditions (pipette solution containing Na^+^ and Glutamate; see Materials and Methods). After adding 10 mM test divalent cations to the NMDG^+^ solution, we recorded currents using step voltages from two holding potentials (-60 mV and +40 mV). We determined G185R-specific currents by measuring the reversal potentials of the currents subtracted from two holding potentials (see Figure 4A and 4B) and corrected for the junction potential. The permeability sequence was Ca^2+^ > Sr^2+^ > Ba^2+^ as calculated (Equation 2; see Materials and Methods) and illustrated in Figure 3E. For divalent cations, we found that the highest conductance was to Ca^2+^, followed by Sr^2+^ and Ba^2+^ (Figure 3D). While Ca^2+^, Sr^2+^, and Ba^2+^ currents were relatively stable over time, currents mediated by Mn^2+^ and Mg^2+^ were transient (Figure 3D), the simplest explanation for this behavior being a block by these two weakly permeant ions. The monovalent permeability was calculated using Equation 1 (see Materials and Methods), yielding a selectivity sequence Li^+^ > Na^+^ > K^+^ > Cs^+^ (Figure 3E). Under these conditions, P_Mn _was insignificant. The cationic permeability sequence (Figure 3E) of I_G185R_ was similar to L-type voltage-gated Ca^2+^ channels (VGCCs) (Sather and McCleskey 2003), but I_G185R_ was less Ca^2+^-selective (P_Ca_/P_Na _of approximately 10) than VGCCs (P_Ca_/P_Na _of approximately 1,000). Single *i*_G185R_ channels were not observed in cell-attached patches. Analysis of membrane current noise at −100 mV predicted a single-channel chord conductance of 0.4 ± 0.1 pS (*n* = 5; unpublished data), too small to be observed under most patch–clamp conditions.

Using the Ca^2+^ indicator dye Fura-2, we demonstrated G185R-mediated Ca^2+^ influx by monitoring intracellular Ca^2+^ levels in response to an elevation of [Ca^2+^]_o_ (Figure 3F). To minimize the contributions of endogenous Ca^2+^-influx and/or store release, we bathed cells in the presence of 50 μM SKF96365 and 50 μM 2-APB. Upon raising [Ca^2+^]_o_, [Ca^2+^]_i_ rose from 105 nM to 240 nM in doxycycline-induced G185R-HEK-On cells, significantly higher than in control HEK-On cells treated with doxycycline. Thus, the permeability of G185R to Ca^2+^ is capable of increasing [Ca^2+^]_i_.

I_G185R_ displayed hyperpolarization-induced inhibition (Figure 4A and 4B) (Bakowski and Parekh 2000). The half-maximal activation voltages (V_1/2_) were −33 mV and −10 mV for control and isotonic Ca^2+^ solutions, respectively (Figure 4B). The voltage-dependence of I_G185R_ was Ca^2+^-independent, since the Na^+^ and Li^+^ currents in nominal [Ca^2+^]_o_ also exhibited a similar voltage dependence. By contrast, I_DMT1_ lacked this voltage dependence (Figure 4B). I_G185R_ was not enhanced under low-divalent conditions (less than 10 nM), nor was it blocked by antagonists of known Ca^2+^-permeant channels. In particular, the current was not blocked by ruthenium red (RR), Ca^2+^-release activated Ca^2+^ channel (CRAC) blockers SKF96365 and 2-APB (Kozak et al. 2002; Prakriya and Lewis 2002) (Figure 4C), or the L-type VGCC blocker nifedepine (10 μM). Divalent cations, including DMT1 substrates (Cd^2+^, Ni^2+^, Co^2+^), inhibited I_G185R_. La^3+^ (1 mM; Figure 4C) and Cd^2+^ (1 mM) blocked I_G185R_ in a similar voltage-dependent manner (Figure 4D). Thus, I_G185R_ is distinct from known Ca^2+^-permeant channels such as VGCCs, transient receptor potentials (TRPs), and CRAC currents, based on its current–voltage (I-V) relation, kinetics, permeation properties, and pharmacological sensitivity.

**Figure 5:**
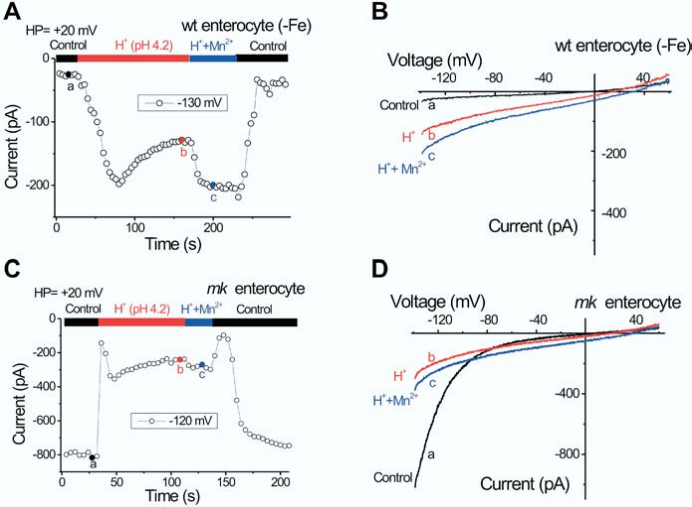
DMT1-Like and G185R-Like Currents in Enterocytes Isolated from Wild-Type and *mk*/*mk* Mice, Respectively (A) Enterocyte currents isolated from an iron-deficient wild-type mouse (−Fe). Reducing bath pH (140 mM NaCl) induced a slowly desensitizing inward current that was further enhanced by addition of Mn^2+^. (B) Both proton and H^+^/Mn^2+^currents were inwardly rectifying. (C and D) An *mk* enterocyte expressed a large constitutive inward current in control bath solution. Reducing the bath pH (140 mM NaCl) first inhibited and then activated another inward current insensitive to the holding potential. This slowly-desensitizing current displayed a less steeply rectifying I-V as shown in (D).

To investigate whether G185R-induced Ca^2+^ permeability might play a physiological role in the mutant animals, we recorded from intestinal enterocytes isolated from both wild-type and homozygous *mk* mice. We studied cells from the proximal 1 cm of the mouse duodenum, where DMT1 expression is highest and iron absorption is maximal (Gunshin et al. 1997; Canonne-Hergaux et al. 1999). Because DMT1 expression is very low in iron-replete, wild-type mice, but induced in iron-deficient mice (Canonne-Hergaux et al. 1999), we isolated enterocytes from mice that had been made iron-deficient by prolonged feeding of an iron-deficient diet, and confirmed DMT1 induction by Western blotting using a DMT1-specific antibody (unpublished data). We were able to record I_DMT1_-like currents in mature enterocytes that stained positive for alkaline phosphatase (I > 80 pA at −130mV, *n* = 7 out of 20 cells; representative data shown in Figure 5A and 5B).

Mice homozygous for the *mk* mutation express large amounts of G185R DMT1 protein in the duodenum. Although much of it is mislocalized to the cytoplasm (Canonne-Hergaux et al. 2000), we expected that some would be present in the plasma membrane. Accordingly, and in contrast with wild-type enterocytes, we recorded a large, constitutive inward current in most mature *mk* enterocytes (*n*= 6 out of 8 cells; Figure 5C and 5D), which displayed the same conductance as seen in G185R-transfected cells. The I-V relationship, step current response, dependence on holding potential, ion selectivity and insensitivity to RR, and SKF96365 or 2-APB were indistinguishable from those of transfected I_G185R_. Furthermore, H^+^ inhibited the I_G185R_-like current in *mk* enterocytes, and the H^+^/Mn^2+^-induced DMT1-like current at pH 4.2 (Figure 5C and 5D) was insensitive to holding potential, as observed in transfected cells. Based on these observations, we conclude that the major current observed in *mk* enterocytes was I_G185R_. Although our preparation did not allow us to distinguish apical versus basolateral localization, the large size of the current in *mk* cells was consistent with plasma membrane localization of G185R protein.

## Discussion

We conclude that expression of G185R in transfected cells and in vivo in *mk* mice is associated with the appearance of a novel Ca^2+^ permeation pathway that has the properties of a Ca^2+^ channel. One interpretation is that a Ca^2+^ channel pathway through the DMT1 protein is exposed or augmented by the G185R mutation. Another possibility is that Ca^2+^ conduction occurs through an associated Ca^2+^-permeable protein. We favor the first possibility because the Ca^2+^ conductance has been observed in diverse cell lines expressing G185R DMT1 (CHO-K1, HEK293T, and HEK-On cell lines) and in *mk* enterocytes. A putative associated protein, if present in these different cell types, would have to be activated in a G185R-dependent manner. We did not find evidence of an associated protein when we immunoprecipitated wild-type or G185R DMT1 from transfected CHO-K1 cells (unpublished data). Furthermore, a distinct DMT1 mutant, G185K, also displayed Ca^2+^ permeability, but this mutant was less selective for Ca^2+^ over Na^+^ (unpublished data).

G185R mutations have occurred at least three times in rodents, which suggests that G185R not only inactivates DMT1, but may confer an unknown selective advantage. Because it has arisen in inbred colonies, the postulated selective advantage must either make the animals more viable than other *DMT1* mutants with impaired iron transport or more likely to be noticed by those managing the animal colonies. In parallel with these studies, we have generated knockout mice homozygous for a null *DMT1* allele (*Dmt1^–/–^*; H. Gunshin and N. C. Andrews, personal communication). Although detailed phenotypic characterization has not yet been completed, we have noted that *Dmt1^–/–^* mice invariably die by the end of the first week of life, in contrast to *mk/mk* mice, which are poorly viable but can survive for more than a year (H. Gunshin and N. C. Andrews, personal communication). This suggests that the small amount of residual function of G185R DMT1, perhaps in combination with its gain-of-function Ca^2+^ conductance, contributes to viability.

Two previous studies support the notion that the gain-of-function reported here is an advantage. Elevated intracellular [Ca^2+^] has been reported to increase nontransferrin-bound iron uptake through an undefined transport system that has characteristics distinct from DMT1 (Kaplan et al. 1991). This might ameliorate the iron-transport defect caused by inactivation of DMT1, either in the intestine or in erythroid precursors. The transferrin cycle is essential for iron uptake by erythroid precursor cells (Levy et al. 1999), and DMT1 mediates at least some of the transfer of iron from transferrin cycle endosomes to the cytoplasm (Fleming et al. 1998; Gruenheid et al. 1999; Touret et al. 2003). Elevated [Ca^2+^]_i_ has been reported to accelerate iron uptake through the transferrin cycle, apparently through activation of protein kinase C (Ci et al. 2003). Thus, the influx of Ca^2+^ might potentiate the residual DMT1 iron-transport activity. Accordingly, ^55^Fe uptake by *mk/mk* reticulocytes has been reported to be approximately 45% of the level observed in wild-type reticulocytes (Canonne-Hergaux et al. 2001), higher than expected for a severe loss-of-function mutation.

In summary, we have found that a single point mutation (G185R) in a 12-TM transporter protein conferred new Ca^2+^-selective permeability. Previous studies have suggested that channels, pumps, and transporters may share some common mechanisms for ion translocation (Gadsby et al. 1993; Fairman et al. 1995; Cammack and Schwartz 1996; for review see references in Lester and Dougherty 1998; Nelson et al. 2002). The “channel mode” has been proposed to explain the “drive-slip” mechanism as part of the transport cycle. In this sense, wild-type DMT1 may simply be a proton channel with limited permeability for certain divalent metal ions. By mutating a single residue, G185R, it becomes an unambiguously Ca^2+^-permeant ion channel. Our findings may add new insight into mechanisms of Ca^2+^ entry and transporter function. The notion that the 12-TM proteins can be ion channels may inform the search for candidate Ca^2+^ and/or cationic channels and facilitate the molecular characterization of many unidentified native conductances.

We initiated these studies to investigate why a unique DMT1 mutation, G185R, has occurred independently at least twice in mice and once in rats (Fleming et al. 1997,^1998^). The multiple occurrences of this spontaneous mutation suggested that it might confer some type of selective advantage. We speculate that the proposed Ca^2+^ entry gain of function helps to account for this remarkable pattern of remutation. Further investigation of this hypothesis will require direct and detailed comparison of *DMT1*-null and *mk* mice.

## Materials and Methods

### 

#### Molecular biology

The *DMT1* cDNA used in this study was derived from one of four alternatively-spliced *DMT1* gene transcripts. The G185R mutation was generated by using M13 phage and the oligonucleotide GTCCCCCTGTGGGGC*C*GAGTCCTCATCACCA. Wild-type DMT1 and the G185R mutant were tagged with a C-terminal FLAG epitope and subcloned into pTracer-CMV2 (Invitrogen, Carlsbad, California, United States). CHO-K1 or HEK293T cells transiently transfected with DMT1 and G185R were used for the ^55^Fe uptake assay and Western blot analysis. To obtain a stable G185R-expressing cell line, the G185R-encoding *DMT1* gene was subcloned into pRevTRE (Clontech, Palo Alto, California, United States), a retroviral vector that drives expression from a Tet-responsive element. All constructs were confirmed by sequencing. DMT1 Western blot analyses were performed with an anti-FLAG M2 monoclonal antibody (Sigma, St. Louis, Missouri, United States) and, in some cases, with a goat polyclonal antibody raised against human DMT1 (Santa Cruz Biotechnology, Santa Cruz, California, United States).

#### Mammalian cell electrophysiology

Wild-type and G185R mutant DMT1 were subcloned into an EGFP-containing vector (pTracer-CMV2, Invitrogen) for transient expression in CHO-K1 and HEK293T cells. Cells were transfected using Lipofectamine 2000 (Invitrogen). Transfected cells, cultured at 37°C, were plated onto glass coverslips and recorded 24 (DMT1) or 30 (G185R) hrs after transfection. A stable cell line (HEK293 Tet-On^TM^, or HEK-On) was generated, and expression was induced by adding 1–10 μg/ml doxycycline into the culture medium. Unless otherwise stated, the pipette solution contained 147 mM cesium, 120 mM methane-sulfonate, 8 mM NaCl, 10 mM EGTA, 2 mM Mg-ATP, 20 mM HEPES (pH 7.4). Bath solution contained 140 mM NaCl, 10 mM CaCl_2_, 10 mM HEPES, 10 mM MES, 10 mM glucose (pH 7.4). Unless otherwise stated, the low pH solutions contained only nominal free Ca^2+^ (1–10 μM). Data were collected using an Axopatch 2A patch–clamp amplifier, Digidata 1320, and pClamp 8.0 software (Axon Instruments, Union City, California, United States). Whole-cell currents were digitized at 10 kHz and filtered at 2 kHz.

The permeability to monovalent cations (relative to P_Na_) was estimated according to Equation 1 from the shift in E_rev_ upon replacing [Na^+^]_o_ in nominally Ca^2+^-free bath solution (150 mM XCl, 20 mM HEPES, 10 mM glucose [pH 7.4]]), where X^+^ was Na^+^, K^+^, Cs^+^, or Li^+^. For the permeability to divalent cations (relative to P_Na_), bi-ionic conditions were used; Y^2+^ was Ca^2+^, Ba^2+^, or Sr^2+^ (Equation 2). The internal pipette solution contained 100 mM Na-gluconate, 10 mM NaCl, 10 mM EGTA, 20 mM HEPES-Na (pH 7.4 adjusted with NaOH, [Na^+^]_total_ = 140). The external solution was 140 mM NMDG-Cl, 10 mM Y^2+^Cl_2_, 20 mM HEPES (pH 7.4 adjusted with HCl). The permeability ratios of cations were estimated from the following equations (Lewis 1979):



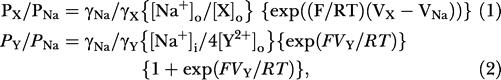



where R, T, F, V, and γ are, respectively, the gas constant, absolute temperature, Faraday constant, E_rev_, and activity coefficient. The liquid junction potentials were measured and corrected as described by Neher (1992).

#### Uptake assay

The assay buffer contained 25 mM Tris, 25 mM MES, 140 mM NaCl, 5.4 mM KCl, 5 mM glucose, 1.8 mM CaCl_2_, 0.8 mM MgCl_2_. Ascorbic acid was adjusted to 1 mM and the pH was adjusted to 5.8. Most assays were performed with 20 μM Fe^2+^ at pH 5.8 unless otherwise indicated. A 50-fold ^55^Fe stock was made immediately before the assay with 1 mM ^55^Fe (with a 1:20 molar ratio for ^55^FeCl_3_ and FeSO_4_) and 50 mM nitrilotriacetic acid. About 30 h after transient transfection, CHO-K1 or HEK293T cells were washed and harvested with PBS (for CHO-K1 cells, trypsin treatment was required). Cells were resuspended in glass test tubes at 0.5–1 million/ml in 490 μl assay buffer at 30°C. The reaction was started by adding 10 μl of ^55^Fe stock and stopped at 4, 8, and 16 min by quickly filtering the reaction mix on a nitrocellulose filter (HAWP02500; Millipore, Billerica, Massachusetts, United States). Filters were washed twice with 2 ml of assay buffer, dried, and radioactivity counted by liquid scintillation spectrometry.

#### Calcium imaging

Cells were loaded with 2 μM Fura-2 AM in culture medium at 37°C for 30 min. Low levels of G185R protein were expressed in the absence of doxycycline in G185R HEK-On cells (Western blotting; unpublished data). Therefore, doxycycline-treated HEK-On cells not expressing DMT1 were used as controls in imaging experiments. We recorded Fura-2 ratios (F340/F380) on an UltraVIEW imaging system (Olympus, Tokyo, Japan). A standard curve for Fura-2 ratio versus [Ca^2+^] was constructed according to Grynkiewicz et al. (1985).

#### Isolation of enterocytes

Homozygous *mk* mice (Fleming et al. 1997) were housed in the barrier facility at Children's Hospital (Boston, Massachusetts, United States). Husbandry and use were according to protocols approved by the Animal Care and Use Committee. Wild-type iron-deficient mice were provided by J.-J. Chen (Massachusetts Institute of Technology, Cambridge, Massachusetts, United States). Mouse enterocytes were isolated using a modified protocol provided by Dr. F. Sepulveda (Monaghan et al. 1997). In brief, 1 cm of the proximal duodenum was excised, rinsed with cold PBS, and soaked for 5 min at 37°C in a solution containing 7 mM K_2_SO_4_, 44 mM K_2_HPO_4_, 9 mM NaHCO_3_, 15 mM Na_3_Citrate, 10 mM HEPES, and 180 mM glucose (pH 7.4). The tissue was then incubated with gentle shaking for 3 min in a similar solution containing 7 mM K_2_SO_4_, 44 mM K_2_HPO_4_, 9 mM NaHCO_3_, 10 mM HEPES, 180 mM glucose, 1 mM DTT, and 0.2 mM EDTA (pH 7.4). The mucosal cells were gently squeezed from the duodenum with forceps into 5 ml of ice-cold DMEM/F12 medium, pelleted at 800 × *g* for 4 min, resuspended in 5 ml of prewarmed DMEM/F12 with 0.5 mg/ml collagenase type 1A, and incubated at 37°C for 10 min. Cells isolated by this procedure have been shown previously to be primarily of villus origin and hence are mature enterocytes. We confirmed this by alkaline phosphatase staining. Diluted cells were filtered through a 40-μm nylon cell mesh (BD Biosciences, Palo Alto, California, United States). The cells were then washed with DMEM/F12, resuspended in 20 ml of ice-cold DMEM and kept at 4°C. They were plated on coverslips coated with Cell-Tak^TM^ (BD Biosciences) and maintained on ice before patch–clamp recording at room temperature.

#### Data analysis

Group data are presented as mean ± SEM. Statistical comparisons were made using analysis of variance and the *t*-test with Bonferroni correction. A two-tailed value of *p* < 0.05 was taken to be statistically significant.

## Supporting Information

Figure S1 CHO-K1 Cells Express an Endogenous Proton-Activated Chloride Channel (A) Anion dependence of pH-induced response in a DMT1-expressing cell. Outward current usually appears later than the inward current.
(B) Currents generated in response to a voltage ramp.
(C) pH-induced outwardly rectifying current in a nontransfected CHO-K1 cell. A similar current was seen also in HEK293T and HEK-On cells, with properties similar to the cloned ClC-7 channel (Diewald et al. 2002). This current exhibits the same anion depen-dence as in (A) (data not shown)**. **We attributed the outward currents shown in (A) and (B) to this endogenous Cl^–^ current. Therefore, for our recordings on DMT1, SO_4_^2\–^ was usually used to replace most of the Cl^–^ ([Cl^–^]_o_ = 5 mM) for all low-pH bath solutions.
(718 KB PDF).

Figure S2 Time- and Voltage-Dependent Kinetics of H^+^/Mn^2+^ Current of DMT1 Whole-cell currents were generated by voltage steps from −140 to +80 mV in 20 mV steps, 400 ms. The interval between steps was 1,000 ms. (1 MB PDF).

Figure S3 Na^+^-Dependence of DMT1 H^+^ and H^+^/Mn^2+^ Currents Replacement of extracellular Na^+^ by NMDG^+^ slightly increased the proton current (approximately 20%) and this was further augmented by adding 300 μM Mn^2+^. The concentrations used were Na^+^ and NMDG^+^, 140 mM, (pH 4.2); Mn^2+^, 300 μM. (141 KB PDF).

### 

#### Accession Numbers

The GenBank (www.ncbi.nlm.nih.gov/GenBank/) accession number for *DMT1* is AF029758.

## Abbreviations

CRAC: Ca^2+^-release activated Ca^2+^ channel
DIDS: diisothiocyanostilbene 2,2-disolphonic acid
DMT1: divalent metal transporter-1
EGFP: enhanced green fluorescent protein
E_rev_: reversal potential
I_G185R_: inward current mediated by the G185R mutant DMT1
I-V: current–voltage
*mk*: microcytic
NMDG: *N*-methyl-D-glucamine
RR: ruthenium red
TM: transmembrane
TRP: transient receptor potential
VGCC: voltage-gated Ca^2+^ channel
