# Supplementary material for: A Spontaneous, Recurrent Mutation in Divalent Metal Transporter-1 Exposes a Calcium Entry Pathway
Source: PLoS Biol. 2004 Mar 16;2(3):e50. doi: 10.1371/journal.pbio.0020050 (PMC368157; doi:10.1371/journal.pbio.0020050)
Supplement: Figure S1 — (A) Anion dependence of pH-induced response in a DMT1-expressing cell. Outward current usually appears later than the inward current. (B) Currents generated in response to a voltage ramp. (C) pH-induced outwardly rectifying current in a nontransfected CHO-K1 cell. A similar current was seen also in HEK293T and HEK-On cells, with properties similar to the cloned ClC-7 channel (Diewald et al. 2002). This current exhibits the same anion depen-dence as in (A) (data not shown). We attributed the outward currents shown in (A) and (B) to this endogenous Cl– current. Therefore, for our recordings on DMT1, SO4 2\– was usually used to replace most of the Cl– ([Cl–]o = 5 mM) for all low-pH bath solutions. (718 KB PDF). [file pbio.0020050.sg001.pdf]

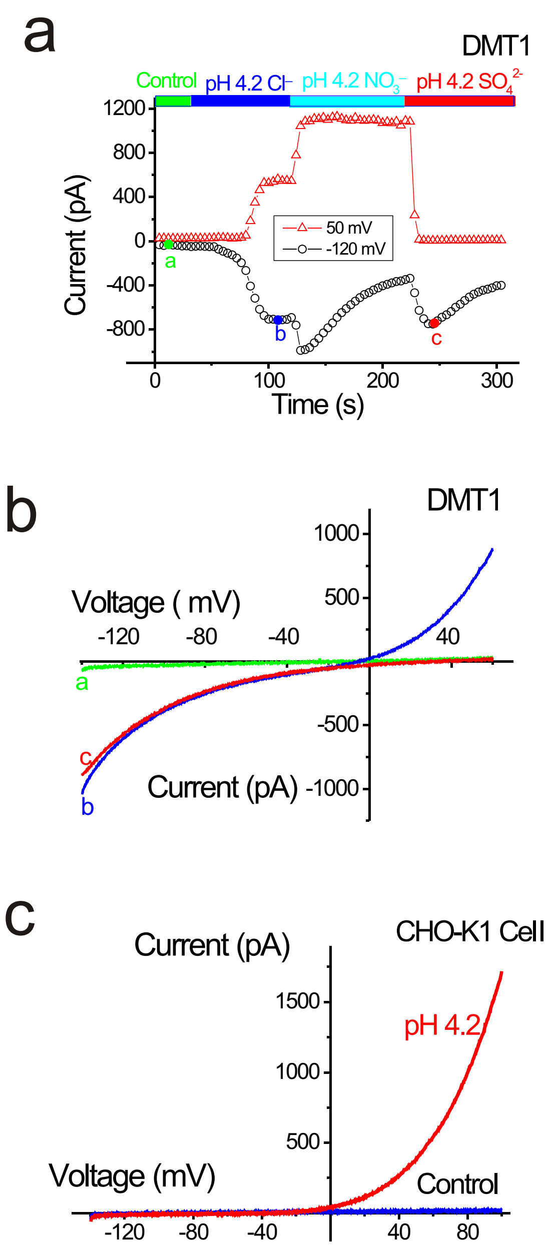

Supplementary Fig. 1. CHO-K1 cells express an endogenous proton-activated chloride channel. **a,** Anion dependence of pH-induced response in a DMT1-expressing cell. Outward current usually appears later than the inward current . **b**, Currents generated in response to a voltage ramp. **c**. pH-induced outwardly rectifying current in a non-transfected CHO-K1 cell. A similar current was seen also in HEK293T and HEK-On cells, with properties similar to the cloned ClC-7 channel (Diewald et al. Activation by acidic pH of CLC-7 expressed in oocytes from *Xenopus* laevis. Biochem Biophys Res Commun. 2002;291(2):421-4.).. This current exhibits the same anion dependence as in **a** (not shown)**.** We attributed the outward currents shown in **a** and **b** to this endogenous Cl^- current. Therefore, for our recordings on DMT1, $SO4^{2-}$ was usually used to replace most of the Cl^- ($[Cl^-]_o$=5 mM) for all low-pH bath solutions.
