# Supplementary material for: A Spontaneous, Recurrent Mutation in Divalent Metal Transporter-1 Exposes a Calcium Entry Pathway
Source: PLoS Biol. 2004 Mar 16;2(3):e50. doi: 10.1371/journal.pbio.0020050 (PMC368157; doi:10.1371/journal.pbio.0020050)
Supplement: Figure S2 — Whole-cell currents were generated by voltage steps from −140 to +80 mV in 20 mV steps, 400 ms. The interval between steps was 1,000 ms. (1 MB PDF). [file pbio.0020050.sg002.pdf]

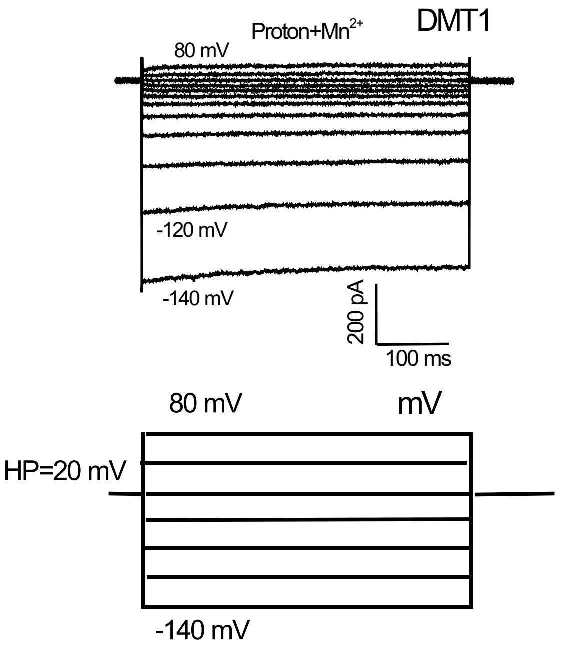

Supplementary Fig. 2. Time and voltage-dependent kinetics of H^{+}/Mn^{2+} current of DMT1. Whole-cell currents were generated by voltage steps from -140 to 80 mV in 20 mV steps, 400ms. The interval between steps was 1000 ms.
