# Supplementary material for: A Spontaneous, Recurrent Mutation in Divalent Metal Transporter-1 Exposes a Calcium Entry Pathway
Source: PLoS Biol. 2004 Mar 16;2(3):e50. doi: 10.1371/journal.pbio.0020050 (PMC368157; doi:10.1371/journal.pbio.0020050)
Supplement: Figure S3 — Replacement of extracellular Na+ by NMDG+ slightly increased the proton current (approximately 20%) and this was further augmented by adding 300 μM Mn2+. The concentrations used were Na+ and NMDG+, 140 mM, (pH 4.2); Mn2+, 300 μM. (141 KB PDF). [file pbio.0020050.sg003.pdf]

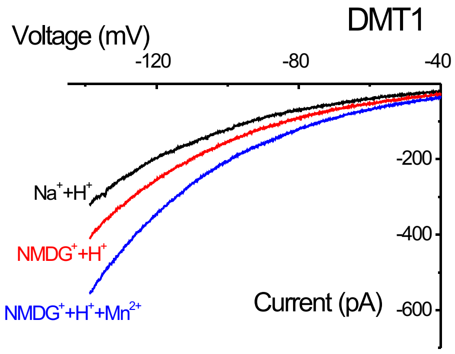

Supplementary Fig. 3. Na^{+}-dependence of DMT1 H^{+} and H^{+}/Mn^{2+} currents. Replacement of extracellular Na^{+} by $NMDG^{+}$ slightly increased the proton current (~20 %) and this was further augmented by adding 300 µM Mn^{2+}. The concentrations used were: Na^{+} and $NMDG^{+}$, 140 mM, pH 4.2, Mn^{2+}, 300 µM.
